# Molecular mechanism of ethanol fermentation inhibition via protein tyrosine nitration of pyruvate decarboxylase by reactive nitrogen species in yeast

**DOI:** 10.1038/s41598-022-08568-4

**Published:** 2022-03-18

**Authors:** Supapid Eknikom, Ryo Nasuno, Hiroshi Takagi

**Affiliations:** Division of Biological Science, Graduate School of Science and Technology, Nara Institute of Science and Technology, 8916-5, Takayama-cho, Ikoma, Nara, 630-0192 Japan

**Keywords:** Biochemistry, Microbiology, Physiology

## Abstract

Protein tyrosine nitration (PTN), in which tyrosine (Tyr) residues on proteins are converted into 3-nitrotyrosine (NT), is one of the post-translational modifications mediated by reactive nitrogen species (RNS). Many recent studies have reported that PTN contributed to signaling systems by altering the structures and/or functions of proteins. This study aimed to investigate connections between PTN and the inhibitory effect of nitrite-derived RNS on fermentation ability using the yeast *Saccharomyces cerevisiae*. The results indicated that RNS inhibited the ethanol production of yeast cells with increased intracellular pyruvate content. We also found that RNS decreased the activities of pyruvate decarboxylase (PDC) as a critical enzyme involved in ethanol production. Our proteomic analysis revealed that the main PDC isozyme Pdc1 underwent the PTN modification at Tyr38, Tyr157, and Tyr344. The biochemical analysis using the recombinant purified Pdc1 enzyme indicated that PTN at Tyr157 or Tyr344 significantly reduced the Pdc1 activity. Interestingly, the substitution of Tyr157 or Tyr344 to phenylalanine, which is no longer converted into NT, recovered the ethanol production under the RNS treatment conditions. These findings suggest that nitrite impairs the fermentation ability of yeast by inhibiting the Pdc1 activity via its PTN modification at Tyr157 and Tyr344 of Pdc1.

## Introduction

Nitric oxide (NO·) is a highly permeable free radical produced in a wide range of cells and tissues. NO• is now known to regulate various biological functions in cells when present at low or appropriate concentrations, including neurotransmission, gene expression, protein translation, apoptosis, and signal transduction^1–4^. In terms of industrial applications, the enhancement of NO• production has previously been reported to improve stress tolerance and fermentation ability in baker’s yeast^5^. Exogenous NO· also increases an oxidative stress response involving a negative feedback system to regulate the intracellular ROS levels in the fission yeast *Schizosaccharomyces pombe*^6^. However, at high concentrations, NO· and various NO-derived molecules known as reactive nitrogen species (RNS) can cause cellular dysfunctions and/or cell death via a process known as nitrosative stress^7^. For example, Almeida and co-workers reported that NO-induced cell death was mediated by *S*-nitrosation of GAPDH as an apoptotic trigger^8^.

After translation, proteins can be edited enzymatically or non-enzymatically by post-translational modifications (PTMs), which dynamically alter the structures, stabilities, and functions of proteins^9^. Protein tyrosine nitration (PTN) is one of the RNS-dependent PTMs. PTN is a chemical process that selectively introduces a nitro group (NO_2_) to an ortho carbon to a phenolic hydroxyl group on the aromatic ring of a tyrosine (Tyr) residue, leading to the conversion of Tyr into 3-nitrotyrosine (NT)^10^. PTN is caused by several combinatorial reactions of RNS with reactive oxygen species (ROS)^11^, including the production of peroxynitrite (ONOO^­^)^12^. The protein structure, nitration mechanism, and cellular environment of the protein all can influence the biological specificity of PTN^13^. PTN contributes to cellular signaling mechanisms because of its specificity, reversibility, and controlled rate^14,15^. It affects protein structures and functions by the steric hindrance of its nitro group, the deprotonated phenolic hydroxyl group caused by its reduced p*K*a value, or the crosstalk with Tyr phosphorylation signaling, leading to an immune response modulation^16^. It also plays a role in the pathogenesis of inflammatory responses, cytoskeletal dysfunction, platelet activation, age-related macular degeneration (AMD), and neurodegenerative disorders^11,17–21^. There have been few reports about the demodification of PTN. However, a previous study showed that an NT-containing peptide was reduced to a peptide containing 3-aminotyrosine (AT), which is a reduced form of NT, in cells, probably through enzymatic activities^22^. Numerous studies on PTN in mammals and plants have been conducted. However, the NO· signaling mechanisms mediated by PTN in the lower eukaryotic yeasts have remained unclear. A previous study identified yeast mitochondrial proteins including proteins in the TCA cycle and respiratory system, such as aconitase and isocitrate dehydrogenase, as a target of PTN^23^. Furthermore, 14 nitrated proteins have been identified by proteomic analysis using the yeast *Saccharomyces cerevisiae* treated with a mating pheromone; these include proteins related to gene expression, actin cytoskeleton organization, cell wall organization, and spermidine transport^24^. However, the effects of PTN on these yeast proteins have not been investigated.

*S. cereivisae* has been used in the fermentation processes to produce various goods, including alcoholic beverages, breads, and biofuels. When yeast cells promote fermentation to synthesize ethanol, sugars are converted into pyruvate via the glycolytic pathway and then further metabolized to ethanol by the two additional enzymatic reactions, decarboxylation of pyruvate to acetaldehyde by pyruvate decarboxylase (PDC) and reduction of acetaldehyde to ethanol by alcohol dehydrogenase (ADH)^25^. The ethanol production is crucial for oxidation of NADH to maintain the appropriate NAD^+^/NADH redox balance for glycolysis. *S. cerevisiae* cells prefer the fermentative pathway when the glucose concentration is above 150 mg/L to the respiration via the TCA cycle even under aerobic conditions, which is known as the Crabtree effect^26,27^. Only a small amount of acetaldehyde is converted into acetate by aldehyde dehydrogenase^28^. PDC is a cytosolic enzyme that catalyzes the conversion of pyruvate into acetaldehyde and carbon dioxide with thiamin diphosphate (TPP) and Mg^2+^ as cofactors^29^. PDC plays an essential role in the diversion of pyruvate toward fermentation and lipid synthesis in some bacteria, yeasts, fungi, and plants. The genome of *S. cerevisiae* contains three genes encoding PDC, *PDC1*, *PDC5*, and *PDC6*. Pdc1, a major isozyme consisting of 563 amino acids, is highly expressed under most physiological conditions^30^. The catalytically active enzyme in yeast is composed of four identical subunits of Pdc1; however, the dimer is required as the smallest catalytically active unit^31^. Pdc1 in *S. cerevisiae* represents the allosteric regulation of catalytic activity, in which its substrate pyruvate binds to the regulatory site to induce the protein conformational changes^32^.

Molasses is a viscous by-product of the processing of sugar cane or sugar beets into sugar. It has been widely used as one of the most cost-effective carbon sources to produce ethanol and other fermentative products in the industry. In some types of molasses, such as beet molasses, the toxic amounts of organic or inorganic compounds inhibit the rate of ethanol production. Also, molasses contains various amounts of nitrate and nitrite. A previous study showed that the concentration of nitrite, which has been identified as a yeast fermentation inhibitor, in undiluted beet molasses was around 9 mM^33^. Nitrite dramatically induces dose-dependent growth inhibition, the decreased ATP levels, and the inhibition of some enzyme activities, such as glyceraldehyde-3-phosphate dehydrogenase and glutamate dehydrogenase, when present at concentrations above 0.65 mM with pH below 5.0^34–37^. The combination of an acidic condition and nitrite, called acidified nitrite, results in RNS production. Nitrous acid, a protonated form of nitrite under an acidic condition, is converted into dinitrogen trioxide, followed by heterolysis to NO· and nitrogen dioxide radicals^38^. In contrast, the pH of the culture medium during fermentation with yeast using glucose can fall below pH 2^37^. These results suggest that the inhibitory effect of nitrite during ethanol production is caused by nitrosative stress derived from acidified nitrite.

In this study, we analyzed PTN and its effect on ethanol production in *S. cerevisiae* cells under nitrosative stress conditions induced by acidified nitrite. Our findings show that *S. cerevisiae* cells produced less ethanol, which was caused by the decreased Pdc1 activity via PTN of Pdc1 at specific Tyr residues, when exposed to nitrosative stress. These findings provide new insights into the molecular mechanism underlying the negative effect of nitrite in medium on ethanol synthesis via the RNS-mediated PTN.

## Results

### RNS produced from acidified nitrite decreases ethanol production and PDC activity

**Figure 1:**
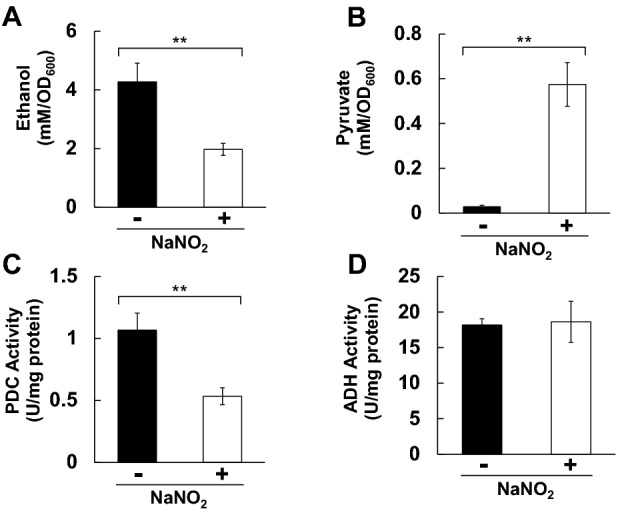
Effect of acidified nitrite on ethanol production and the related enzymatic activity. (**A**) Ethanol concentration in the culture medium, or (**B**) intracellular pyruvate content of yeast with or without acidified nitrite treatment was analyzed. Each content was normalized by OD_600_ of the culture medium. (**C**) PDC, or (**D**) ADH activity in the cell-free extract from yeast exposed to acidified nitrite was measured. The values represent the averages and standard deviations from three independent experiments. **ρ < 0.01 by Student’s *t*-test.

We first examined the effect of nitrosative stress on ethanol production in *S. cerevisiae*. When yeast cells were treated with 1 mM NaNO_2_ at pH 4.0 for 1 h, the ethanol concentration in culture medium was significantly lower than that of untreated cells (Fig. 1A). This result indicates that nitrosative stress inhibits ethanol production, consistent with the previous study^33^. Subsequently, the intracellular concentration of pyruvate, the end product of glycolysis, was measured. Interestingly, the pyruvate content dramatically increased when yeast cells were treated with acidified nitrite (Fig. 1B). To investigate the mechanism by which RNS generated from acidified nitrite decreases the ethanol production ability, we measured the activity of the enzymes involved in the ethanol synthetic pathway from glycolysis, PDC and ADH. Importantly, the PDC activities in the total protein extracts from the cells treated with acidified nitrite were approximately 50% lower than those from untreated cells (Fig. 1C), although ADH was fully active regardless of stress treatment (Fig. 1D). These results suggest that PDC activity was specifically decreased by RNS, resulting in the lower ethanol content.

### Pdc1 is the target of PTN at positions Tyr38, Tyr 157, and Tyr344

Pdc1, the main isozyme of three PDCs in *S. cerevisiae*^39^, was further analyzed as a protein responsible for the decreased PDC activity under nitrosative stress conditions. Western blotting analysis revealed that nitrite treatment did not change the protein level of Pdc1 (Fig. S1). This result raised the possibility that PTMs of Pdc1 are involved in the decrease of its activity. In addition, we subjected the total protein extract from cells treated with acidified nitrite to a proteomic analysis using the stable isotope labeling with amino acids in cell culture (SILAC) method^40^. As a result, we identified Pdc1 with 76.02% of the protein sequence coverage, including 14 of the 17 Tyr residues of Pdc1 (Fig. S2, Table 1). Among the peptides annotated as Pdc1, no peptides containing NT but some peptides containing AT were detected, suggesting that the intracellular reduction of NT occurred as previously reported^22^. Therefore, we concluded that these AT-containing peptides were derived from Pdc1 with PTN. From these results, we detected three AT-containing peptides in the sample treated with nitrite, which respectively contained PTN at Tyr38, Tyr157, or Tyr344, although no quantitative information was available because the signals of AT-containing peptides were detected in only either the control or nitrite-treated samples (Tables 1 and S1–S3, Fig. S3). The raw data of proteomic analysis were deposited to a public repository Japan ProteOme STandard Repository (jPOSTrepo, https://repository.jpostdb.org/) and available with the accession number of JPST001473^41^.

**Table 1:** Identified peptides of Pdc1 containing trace of PTN in the proteomic analysis.

Peptide sequence	AT modification site
QVNVNTVFGLPGDFNLSLLDKIY*EVEGMR	Tyr38
T^ǂ^TY*VTQR^#^PVYLGLPANLVDLNVPAK^#^	Tyr157
G^ǂ^Y*K^#^PVAVPAR^#^	Tyr344

Y* = Tyr residues detected as AT, # = SILAC labeling of Lys (K) and Arg (R), ǂ = N-terminal acetylation.

**Figure 2:**
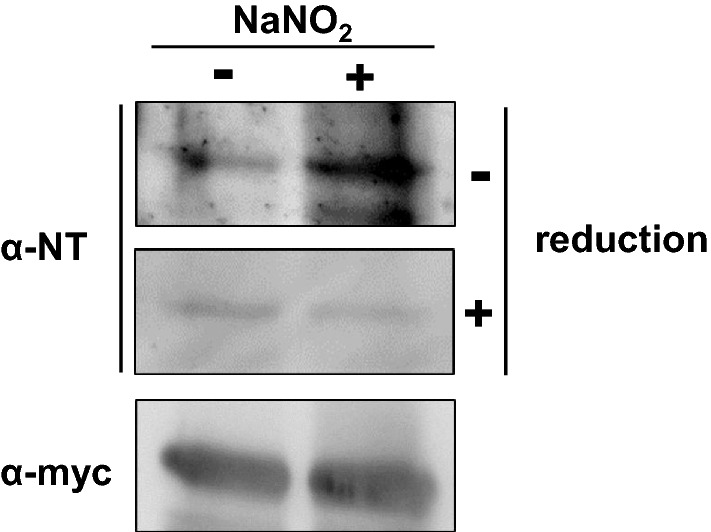
PTN modification of Pdc1 in response to RNS. The PDC1-myc7His strain grown until the exponential phase was treated with acidified nitrite and then the extracted lysate was subjected to pull-down assay, followed by western blot analysis with anti-NT or anti-myc antibody. The PVDF membrane was incubated with sodium dithionite before the treatment with anti-NT antibody as a negative control for the PTN analysis.

Subsequently, to confirm the PTN modification of Pdc1, yeast cells expressing Pdc1 fused with the myc7His epitope tag at its C-terminus were subjected to a pull-down assay, followed by western blot with anti-NT antibody. The results revealed that an acidified nitrite treatment increased the PTN level of Pdc1, consistent with the above results of proteomic analysis, but the increase in the PTN level was abolished by dithionite reduction (Figs. 2 and S4). These results indicated that Pdc1 was nitrated in response to NO· treatment in yeast cells.

### RNS induce PTN and the inactivation of Pdc1

**Figure 3:**
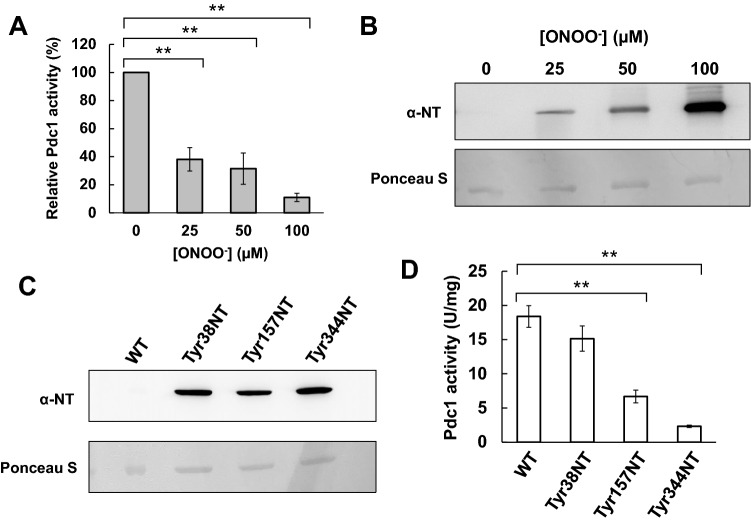
Effect of the PTN modification of Pdc1 on its enzymatic activity. (**A**) The enzymatic activity of recombinant WT-Pdc1 treated with the indicated concentration of ONOO^-^ was measured. The values are the averages and standard deviations from three independent experiments. (**B**) Immunoblotting of WT-Pdc1 treated with various concentrations of ONOO^-^ using anti-NT antibody was shown. The membrane was stained with Ponceau S to confirm the unified protein loaded. (**C**) Incorporation of NT into the recombinant Pdc1 prepared the heterologous expression system in *E. coli* was examined by western blotting with anti-NT antibody. The staining with Ponceau S was used for a loading control. (**D**) The enzymatic activity of WT-, Tyr38NT-, Tyr157NT-, or Tyr344NT-Pdc1 was measured. The values represent the averages and standard deviations from three independent experiments. **ρ < 0.01 by Student’s *t*-test.

A wild-type (WT) enzyme of the recombinant Pdc1 was prepared using a heterologous expression system in *Escherichia coli*. The purified Pdc1 was treated with ONOO^-^ and then its activity was analyzed. The result indicated that the incubation with ONOO^-^ inhibited the enzymatic activity of Pdc1 in a dose-dependent manner (Fig. 3A). Samples prepared by the same method were analyzed by western blot using anti-NT antibody. The results showed that the ONOO^-^ treatment induced dose-dependent increases of the PTN level on Pdc1 without any decrease in the protein amount of Pdc1 (Figs. 3B and S5). These results suggest that the RNS-dependent PTN inactivates Pdc1.

### PTN at Tyr157 or Tyr344 inhibits Pdc1 activity

In order to examine the effect of PTN on the Pdc1 activity at the Tyr residues that were identified as AT in proteomic analysis, the Pdc1 variants with the site-specific incorporation of NT at Tyr38, Tyr157, or Tyr344, which were named Tyr38NT-, Tyr157NT-, or Tyr344NT-Pdc1, respectively, were expressed and purified using the heterologous expression system in *E. coli*. Our immunoblotting analysis with anti-NT antibody showed a clear signal from each Pdc1 variant, indicating that these variants successfully contained NT (Figs. 3C and S6). Next, we measured the enzymatic activities of these Pdc1 variants (Fig. 3D). We found that the activity of Tyr38NT-Pdc1 was almost the same as that of the WT enzyme. Interestingly, Tyr157NT- and Tyr344NT-Pdc1 exhibited dramatically lower activity than that of WT-Pdc1. These results indicate that PTN at Tyr157 or Tyr344 is important for the inhibition of Pdc1 activity.

### Prevention of PTN at Tyr157 or Tyr344 recovers ethanol production under RNS treated conditions

**Figure 4:**
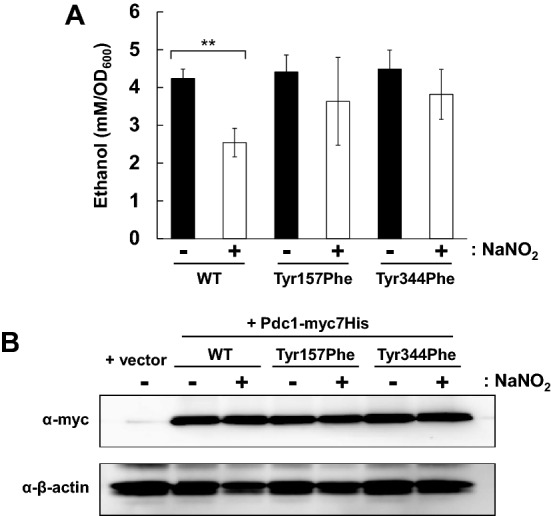
Ethanol production of yeast cells Pdc1 variants resistant to the PTN modification. (**A**) Ethanol contents in the culture medium of yeast producing Pdc1-myc7His with Tyr157Phe or Tyr344Phe substitutions in the presence or absence of acidified nitrite. The values represent the averages and standard deviations from three independent experiments. **ρ < 0.01 by Student’s *t*-test. (**B**) Protein extracts from the cells prepared same as (**A**) were analyzed by western blotting with anti-NT antibody. The *pdc1*Δ *ura3*Δ cells harboring an empty vector pRS416 was used as a negative control.

To clarify the physiological significance of PTN of Pdc1 at each Tyr residue, we further measured the ethanol content in the culture medium of yeast strains. When Tyr157 or Tyr344 was substituted to phenylalanine (Phe) to prevent the PTN formation on these residues, the inhibitory effect of RNS on ethanol production was significantly abolished, although the ethanol content without acidified nitrite treatment was not affected by these substitutions (Fig. 4A). Additionally, it was found that the protein level of Pdc1 was not altered by either acidified nitrite treatment or the amino acid substitution to Phe on each PTN site (Figs. 4B and S7). These results suggest that PTN on Tyr157 and Tyr344 is critical for the inhibition of ethanol production. Therefore, we concluded that the inhibition of Pdc1 activity via its PTN modification at Tyr157 and Tyr344 induced by RNS led to the decreased ethanol productivity in *S. cerevisiae* cells.

## Discussion

Here, we revealed the molecular mechanism by which nitrosative stress affected the ethanol production in yeast cells, which involved PTN of Pdc1 at Tyr157 or Tyr344 inhibiting the enzymatic activity of Pdc1. To the best of our knowledge, this is the first biochemical characterization of the functions of PTN modification on yeast proteins. This study also provides new insights for future research on PTN in yeast.

The previous studies reported that nitrite affected ethanol production and that the culture medium was acidified by prolonged cultivation. Because nitrite is converted into RNS under acidic conditions, the acidified nitrite treatment used in this study could be a model experimental condition to mimic yeast cultivation using molasses, which contains a large amount of nitrite. Our findings clarified the mechanisms by which nitrite impaired the ethanol fermentation of yeast, at least in part. In terms of industrial applications, the yeast strains expressing the Pdc1 variants with Tyr157Phe and Tyr344Phe substitutions are a promising tool to boost ethanol production, because these strains are resistant to the RNS-induced inhibition of ethanol production. Since it is considered that molasses fermentation proceeds under nitrosative stress conditions, such yeast strains may increase the ethanol productivity.

We showed that the intracellular pyruvate content was elevated under nitrosative stress conditions (Fig. 1B). There could be three explanations for pyruvate accumulation under nitrosative stress conditions. First, a decrease in Pdc1 activity causes pyruvate accumulation, as observed in this study. Second, the inhibition of the mitochondrial respiratory chain by the nitrosative stress could induce ATP depletion. The resulting higher AMP/ATP ratio could potentially stimulate glycolysis via the AMP-activated protein kinase to produce ATP, leading to the acceleration of pyruvate synthesis^42^. Third, the pyruvate accumulation could be caused by the impairments of the TCA cycle that consumes pyruvate in mitochondria. The metabolites in glycolysis and the TCA cycle were not measured in this study, and thus it is unsure whether these metabolic pathways were affected by RNS. However, a previous study reported that the activities of some TCA cycle enzymes, including α-ketoglutarate dehydrogenase and pyruvate dehydrogenase, were inhibited by nitric oxide in *Salmonella enterica*^43^. Moreover, it has been reported that the yeast aconitase and NAD^+^-dependent isocitrate dehydrogenase were nitrated in vivo, although the biological significance of these findings has yet to be elucidated^23^. These previous findings imply that pyruvate was accumulated by the inhibition of the TCA cycle enzymes via their PTN modifications, in addition to the inhibition of Pdc1.

Pdc1 is the main isozyme of PDC, which is highly expressed under most conditions^39^ and is also induced by the presence of glucose^44^. Aside from the transcriptional control of *PDC1*, PTMs such as phosphorylation are another means of modulating the function of Pdc1. Dephosphorylation of Pdc1 mediated by the serine/threonine-protein phosphatase increases the ethanol fermentation and the Pdc1 activity by altering the apparent affinity of Pdc1 for TPP and pyruvate in *S. cerevisiae* cells^45^. We did not analyze the phosphorylation status of Pdc1, but it is possible that not only PTN but also the protein phosphorylation are responsible for the control of the Pdc1 enzymatic activity.

Our proteomic analysis identified the peptides containing AT, which is a reduced form of NT, but not those containing NT itself, even though the PTN of Pdc1 was confirmed by immunoblotting analysis (Tables 1 and S1–S9, Figs. 2 and S4). We also did not detect the immonium ion with *m/z* of 181.06, which is a useful footprint of NT-containing peptides, from the peptides assigned to Pdc1^46^. A previous research reported that the PTMs with high electron affinity, such as PTN, prevent the cleavage of the peptide backbone, limiting the sequence information from the MS/MS analysis with electron capture dissociation or electron transfer dissociation^47,48^. On the other hand, it has been reported that proteins with PTN modification were reduced in cells to the corresponding proteins with AT^22^. From this information, it is possible that Pdc1 was nitrated in vivo and then partly reduced to its AT-containing form, which alone was detected in our proteomic analysis.

**Figure 5:**
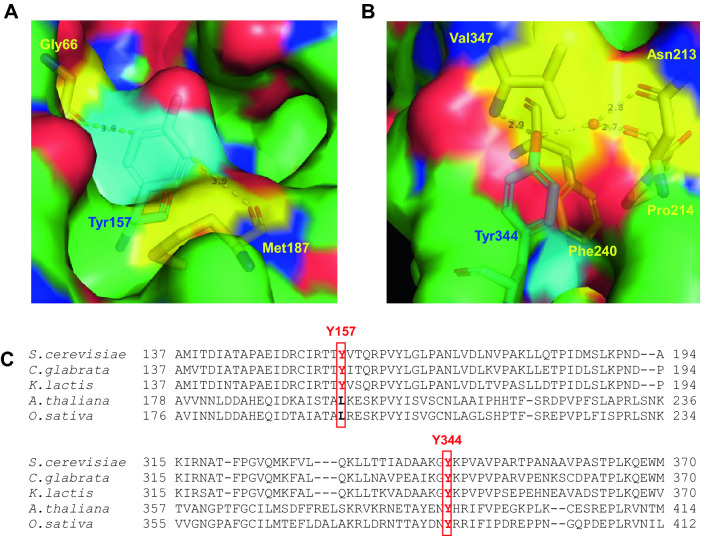
Three-dimensional structure and amino acid sequence conservation of Pdc1. (**A**,** B**) The crystal structures of Pdc1 from *S. cerevisiae* with the PDB ID code of 2VK1^61^ were shown with the molecular surface of protein. Oxygen or nitrogen atom is colored by red or blue. The values nearby dotted lines indicate the distances between two atoms. (**A**) Local structure around Tyr157 was exhibited with Gly66, Tyr157, and Met187 in a stick model. Carbon atoms were colored by cyan, yellow, or green in Tyr157, Gly66 and Met187, or the other residues, respectively. The side chain of Tyr157 is located close to Gly66 and Met187. (**B**) The structure surrounding Tyr344 was shown with Asn213, Pro214, Phe240, Tyr344, and Val347 in a stick model. Carbon atoms were colored by cyan in Tyr344, yellow in Asn213, Pro214, Phe240, and Val347, or green in the other residues, respectively. (**C**) Amino acid sequences of Pdc1 from S. cerevisiae, *Candida glabrata*, *Kluyveromyces lactis*, *Arabidopsis thaliana*, and *Oryza sativa* were aligned using the sequence alignment tool Align in the Universal Protein Resource (UniProt) (https://www.uniprot. org/align/) and the part of alignment was shown. Tyr157 and Tyr344 in Pdc1 from *S. cerevisiae* and their corresponding residues in PDCs from the other species were highlighted by red boxes.

As shown in this study, PTN of Pdc1 at Tyr157 and Tyr344 was responsible for the decrease in the enzymatic activity of Pdc1 (Fig. 3D). The findings in a previous study^49^ suggest that PTN of Pdc1 is likely to contribute to the alteration of the Pdc1 structure and thus its activity. The crystal structure of Pdc1 has been determined at 1.71 Å of resolution in the presence of its substrate pyruvate and cofactor TPP (PDB ID code: 2VK1). In the Pdc1 structure, neither Tyr157 nor Tyr344 directly interacts with the binding site for pyruvate or TPP. On the other hand, because the aromatic ring of Tyr157 is located near Gly66 and Met187, the addition of a nitro group to the side chain of Tyr157 is likely to induce a steric hindrance with these residues (Fig. 5A). Tyr344 is buried inside the protein molecule and interacts with several residues, including Asn213, Pro214, and Val347 or Phe240, through hydrogen bonds or π-π stacking, respectively (Fig. 5B). These intramolecular interactions of Tyr157 or Ty344 imply that the PTN modifications at these sites disturb the correct direct interaction of these Tyr residues, leading to an indirect alteration of the whole structure and enzymatic activity of Pdc1. Our enzyme assay using the site-specifically NT-incorporating Pdc1 exhibited that PTN at either Tyr157 or Tyr344 decreased its activity (Fig. 3D), indicating that both PTN modifications are capable of inhibiting the Pdc1 activity. On the other hand, the ethanol production was recovered by either Tyr157Phe or Tyr344Phe substitution, suggesting that PTN modification at both Tyr residues is required to reduce the ethanol synthesis in yeast cells (Figs. 4B and S7). An additive inhibition of Pdc1 activity by the combined PTN modifications at these two sites might be necessary to inhibit the ethanol production. The Tyr157 and Tyr344 residues are completely conserved among yeast species (Fig. 5C), suggesting that the inhibition of ethanol production by PTN of Pdc1 is a common phenomenon in yeasts. In contrast, Tyr344 is also conserved in plants, although Tyr157 is not. Plants might possess some functions mediated by the PTN modification at Tyr344.

In contrast to our findings, a previous study using 0.5 mM NaNO_2_ for acidified nitrite treatment found that yeast cells exposed to nitrosative stress produced more ethanol in a rich medium by increasing the alcohol dehydrogenase activity under stress conditions, by using 0.5 mM NaNO_2_ for acidified nitrite treatment^50^. Lower concentrations of acidified nitrite could produce lower concentrations of RNS. A milder nitrosative stress might be insufficient to cause Pdc1 nitration but adequate to inhibit the mitochondrial electron transport chain, and then activate glycolysis to produce ATP^51^. Therefore, a lower concentration of nitrite under acidic conditions might increase ethanol production through the enhancement of glycolytic metabolic flow, providing pyruvate a substrate for ethanol synthesis.

The alcohol dehydrogenase involved in ethanol production is an essential enzyme for NAD^+^ regeneration in yeast cells^52^. Our findings here suggest that the nitrosative stress impaired ethanol synthesis by the decreased supply of acetaldehyde through the inhibition of Pdc1. This process could lead to an increase in the NADH/NAD^+^ ratio by inhibiting the oxidation of NADH to NAD^+^ by ADH. NO dioxygenase (NOD) encoded by the *YHB1* gene, which utilizes not only NADPH but also NADH as its reducing force, is the main enzyme in NO· detoxification to nitrate^53^. It is possible that the increased concentration of NADH contributes to the nitrosative stress tolerance via the upregulation of NOD activity.

## Methods

### Strains, plasmids, and medium

Table 2, 3, or S1 represents a list of the strains, plasmids, or primers used in this study, respectively. The yeast *S. cerevisiae* laboratory strain X2180-1A (*MAT*a *SUC2 mal mel gal2 CUP1*) was a host strain to construct the yeast strains used in this study^54^. To generate the strain expressing Pdc1 fused with myc7His-tag at its C-terminus (Pdc1-myc7His) from the genome (the PDC1-myc7His strain), the DNA fragment amplified using the primers listed in Table S1 and plasmid pYM46 (Euroscarf) as a template was introduced into strain X2180-1A by the LiAc method^55,56^. For the construction of strain lacking both the *PDC1* and *URA3* genes (*pdc1*Δ *ura3*Δ), the DNA fragment amplified using primers listed in Table S1 and the plasmid pYM20 as a template was introduced to the strain lacking the *URA3* gene constructed in the previous study^57^. In order to construct the strain lacking the *ARG1* and *LYS1* genes (*arg1*∆ *lys*1∆), which is capable to synthesize neither arginine (Arg) nor lysine (Lys), for the SILAC-based proteomic analysis, strain X2180-1A was transformed with the DNA fragment amplified from plasmid pFA6a-natNT2 (Euroscarf), followed by the further transformation with the fragment from plasmid pFA6a-hphNT1 (Euroscarf), using the primers shown in Table S1. Each positive clone was screened on the solid medium containing antibiotics G418 or hygromycin B. *E. coli* DH5α or BL21 (DE3) strain was used to construct plasmids or express Pdc1, respectively.

**Table 2:** Yeast strains used in this study.

Strains	Genotype
X2180-1A	*MATa SUC2 mal mel gal2 CUP1*
PDC1-myc7His	X2180-1A *PDC1::PDC1*-*myc7His-kanMX6*
*arg1*∆ *lys1*∆	X2180-1A *arg1*∆*::natNT2 lys1*∆*::hphNT1*
WT-PDC1	X2180-1A *ura3*∆*::kanMX4 pdc1*∆*::hph* pRS416*::PDC1-myc7His*
Tyr157Phe-PDC1	X2180-1A *ura3*∆*::kanMX4 pdc1*∆*::hph* pRS416*::PDC1*^*Tyr157Phe*^*-myc7His*
Tyr344Phe-PDC1	X2180-1A *ura3*∆*::kanMX4 pdc1*∆*::hph* pRS416*::PDC1*^*Tyr344Phe*^*-myc7His*

**Table 3:** List of plasmids used in this study.

Plasmids for *S. cerevisiae*	Marker	Type	Promoter	Protein to be produced
pRS416	*URA3*	*CEN*	-	-
pRS416-PDC1-myc7His	*URA3*	*CEN*	*PDC1*	Pdc1-myc7His
pRS416-PDC1^Tyr157Phe^-myc7His	*URA3*	*CEN*	*PDC1*	Pdc1^Tyr157Phe^-myc7His
pRS416-PDC1^Tyr344Phe^-myc7His	*URA3*	*CEN*	*PDC1*	Pdc1^Tyr344Phe^-myc7His

Plasmids for *E. coli*	Marker	Description		
pET55-PDC1	*Amp*^*r*^	To express Pdc1 fused with His-tag at its C-terminus		
pET55-PDC1^Tyr38NT^	*Amp*^*r*^	pET55-DEST to express *PDC1* with amber codon at Tyr38		
pET55-PDC1^Tyr157NT^	*Amp*^*r*^	pET55-DEST to express *PDC1* with amber codon at Tyr157		
pET55-PDC1^Tyr344NT^	*Amp*^*r*^	pET55-DEST to express *PDC1* with amber codon at Tyr344		
pDule-3-nitroTyrosine (5B)	*Tet*^*r*^	To express the *Methanocaldococcus jannaschii* NT-tRNA synthetase and the cognate amber suppressing tRNA for the production of NT-containing protein in *E. coli*		

To construct the plasmid expressing Pdc1-myc7His under the control of its original promoter, the DNA fragment from 1000 bp upstream to downstream of *PDC1* was amplified from the genomic DNA of the PDC1-myc7His strain using the primers listed in Table S1 and then introduced to pRS416 by the In-Fusion cloning system (Clontech) following the manufacturer’s protocol. The resultant plasmid pRS416-PDC1-myc7His was introduced to *pdc1*Δ *ura3*Δ cells, generating the strain expressing Pdc1-myc7His (the WT-PDC1 strain). The expression plasmid pET55-PDC1 used to produce Pdc1 fused with N-terminal strep-tag and C-terminal His-tag in *E. coli* was constructed as follows. The DNA fragment of *PDC1* coding sequence was amplified from the genomic DNA of strain X2180-1A using the primers listed in Table S1 and then introduced to the entry plasmid pDONR221 by the BP reaction in Gateway technology (Invitrogen). The resultant plasmid pDONR221-PDC1 was subjected to the LR reaction with the plasmid pET55-DEST in Gateway technology, generating pET55-PDC1. Each reaction in Gateway technology was performed following the manufacturer’s protocol.

Yeast cells were cultured at 30 °C in YPD (1% yeast extract, 2% peptone, and 2% glucose) or SD (0.17% yeast nitrogen base without amino acids and ammonium sulfate, 0.5% ammonium sulfate, and 2% glucose, pH 4.0) medium, with 200 mg/L of G418, hygromycin B, or 100 mg/mL nourseothricin if necessary. For the SILAC experiment, 206.7 µM Arg and 205.2 µM Lys were supplied. *E. coli* cells were grown in LB medium (0.5% yeast extract, 1% tryptone, and 1% NaCl or M9 medium (0.8% glucose, 47.7 mM Na_2_HPO_4_, 22.0 mM KH_2_PO_4_, 0.05% NaCl, 0.1% NH_4_Cl, 2 mM MgSO_4_, 0.1 mM CaCl_2_, 0.4% casamino acid, and 0.5 mM thiamine), in the presence of 100 μg/mL ampicillin or 10 μg/mL tetracycline when necessary.

### Construction of the plasmid expressing the *PDC1* mutants

The site-directed mutagenesis to express each mutant of *PDC1* was performed as previously described^58^, using the primers listed in Table S1 and pRS416-PDC1 to generate pRS416-PDC1^Tyr157Phe^-myc7His or pRS416-PDC1^Tyr344Phe^-myc7His, which expresses Pdc1-myc7His with Tyr157Phe or Tyr344Phe substitution, respectively. The plasmid pDONR221-PDC1 was used as a template with the primers listed in Table S1 to introduce the amber codon at the position corresponding to Tyr38, Tyr157, or Tyr344. The resultant pDONR221-PDC1 with mutation was subjected to the LR reaction in Gateway technology to generate the pET55-PDC1 plasmids with amber codon, pET55-PDC1^Tyr38NT^, pET55-PDC1^Tyr157NT^, and pET55-PDC1^Tyr344NT^. Successful mutagenesis was confirmed by DNA sequencing.

### Acidified nitrite treatment of yeast

Yeast cells cultured in SD medium (pH 4.0) until the exponential growth phase were treated with 1 mM NaNO_2_ for 1 h at 30 °C. After treatment, cells were collected, frozen with liquid nitrogen, and stored at −80 °C for further analyses.

### PDC and ADH activity

The PDC reaction was performed as previously described^59^. The assay mixture consisted of 40 mM imidazole–HCl buffer (pH 6.5), 0.2 mM thiamine pyrophosphate, 0.15 mM NADH, 88 U/mL alcohol dehydrogenase, 5 mM MgCl_2_, and the cell-free extract or purified Pdc1. The reaction was started by the addition of 50 mM pyruvate. The mixture for ADH reaction consisted of 100 mM phosphate buffer (pH 7.6), 8 mM acetaldehyde, and the cell-free extract. Both enzymatic activities were analyzed spectrophotometrically by monitoring a decrease of absorbance at 340 nm derived from NADH over time at 30 °C, using a spectrophotometer DU-800 (Beckman). One unit of the specific activity of PDC or ADH was defined as the amount of enzyme to oxidize 1 μmol of NADH per min.

### Pull-down assay

After culture or treatment, the PDC1-myc7His cells were collected, washed, and resuspended in the extraction buffer (pH 7.4) containing 20 mM sodium phosphate, 0.5 M NaCl, 8 M urea, 20 mM imidazole, and Protease Inhibitor Cocktail (for Fungal and Yeast) (Wako Pure Chemical Industries), followed by cell disruption using Multi-beads shocker (Yasui Kikai) with glass beads. The supernatant after centrifugation was incubated with His Mag Sepharose™ Ni (Cytiva) equilibrated with the extraction buffer at 4 °C for 1 h with continuous rotation. After washing with the extraction buffer, Pdc1-myc7His was eluted with the elution buffer (pH 7.4) consisting of 20 mM sodium phosphate buffer, 0.5 M NaCl, 8 M urea, and 500 mM imidazole. Eluates were subjected to SDS–polyacrylamide gel electrophoresis (SDS-PAGE), followed by immunoblotting.

### Western blot analysis

Protein concentration was measured by Bio-Rad Protein Assay Dye (Bio-Rad). The unified concentrations of protein extracts were separated by SDS-PAGE under the non-reducing conditions using Laemmli’s sample buffer without 2-mercaptoethanol and then transferred onto PVDF membranes. After the treatment with appropriate primary and secondary antibodies, the immunoblot was visualized using Amersham ECL prime reagents (GE Healthcare) and ImageQuant™ LAS4000 (GE Healthcare). For the negative control of PTN detection, the PVDF membrane was incubated with 10 mM sodium dithionite for 20 min before the treatment with anti-NT antibody. In this study, anti-NT antibody Nitro-Tyrosine Antibody (Cell Signaling Technology, 9691S), anti-myc antibody c-Myc (9E10) (Santa Cruz Biotechnology, sc-40), anti-Pgk1 antibody PGK1 Monoclonal Antibody (22C5D8) (Invitrogen, 459,250), or anti-mouse IgG antibody Anti-Mouse IgG (H + L), HRP Conjugate (Promega, W4021) was used as a primary or secondary antibody, respectively. Some membranes were cut out before the incubation with primary antibodies.

### Expression and purification of WT and NT incorporated Pdc1

In order to express the Pdc1 variants incorporating NT site-specifically, plasmid pET55-PDC1^Tyr38NT^, pET55-PDC1^Tyr157NT^, or pET55-PDC1^Tyr344NT^, which expresses Tyr38NT-, Tyr157NT-, or Tyr344NT-Pdc1, respectively, constructed as described above was used. *E. coli* BL21 (DE3) strain harboring each of these plasmids and the plasmid pDule-3-nitroTyrosine (5B) (Addgene)^60^ were cultured in M9CA medium at 37 °C. When OD_600_ reached 0.6, 0.1 mM isopropyl-β-D-thiogalactopyranoside and 1 mM NT were added and *E. coli* cells were further cultured for 16 h at 16 °C. After harvesting cells by centrifugation, the C-terminally His-tagged Pdc1 was purified by Ni Sepharose™ 6 Fast Flow (Cytiva) following the manufacturer’s protocol.

### Sample preparation for mass spectrometry with SILAC method

Proteomic analysis with SILAC method was performed to identify and quantify the NT-containing peptides. The *arg1*∆*lys*1∆ strain was cultured in SD medium at pH 4.0 containing Arg and Lys or Arg (guanido-^13^C) and Lys (4,4,5,5-D_4_) for the control or nitrite treatment condition, respectively. Cells cultured until the exponential phase were treated with 1 mM NaNO_2_ for 1 h and harvested. The same amount of extracted protein from each sample was mixed and subjected to SDS-PAGE under the non-reducing condition. Sliced gels were treated with 10 mM dithiothreitol at 56 °C for 45 min, 55 mM iodoacetamide at room temperature for 10 min, and then digested with Trypsin Gold, Mass Spectrometry Grade (Promega). The extracted peptides were filtered with a 0.45 μm syringe filter for the following LC–MS/MS analysis.

### LC–MS/MS analysis

A nano-LC systeme ADVANCE UHPLC (AWR) coupled with Ion Trap-Orbitrap Mass Spectrometer LTQ-Orbitrap XL (Thermo Fisher Scientific) was used for LC–MS/MS analysis with a trap column L-column ODS 5 μm (0.5 mm I.D. × 5 mm) (CERI) and a separation column L-column2 ODS 3 μm (0.075 mm I.D. × 150 mm) (CERI). HPLC was performed with a gradient, in which 0.1% formic acid (solvent A) and acetonitrile (solvent B) were used as the mobile phase with a flow rate of 300 nL/min following the program (time, solvent B %); 0 min, 5%; 57 min, 35%; 62 min, 65%; 63 min, 95%; 70 min, 95%; 70.1 min, 5%; and 80 min, 5%. MS/MS analysis was performed with the following parameters, a spray voltage of 2.0 kV, a capillary temperature of 200 °C, and the range of full MS scan with *m/z* from 450 to 1500 with a resolution of 30,000. Fragmentation parameters were as follows; the top three peaks, a collision energy of 35, and a Scan Rate of Normal.

### Database search and analysis

A database search was performed using Proteome Discoverer 1.4 (Thermo Fisher Scientific) with SequestHT node against the Saccharomyces cerevisiae database (NCBI), which was downloaded on May 10, 2019. Searches were done with tryptic specificity allowing maximum 4 missed cleavage sites and a precursor mass tolerance of 10 ppm and fragment mass tolerance of 0.8 Da. Following modifications were searched as variable modifications: Acetyl / + 42.011 Da (Any N-Terminus), Oxidation / + 15.995 Da (M), Carbamidomethyl / + 57.021 Da (C), Nitro / + 44.985 Da (Y), Amino / + 15.011 Da (Y), Label:13C (1) / + 1.003 Da (R), Label:2H (4) / + 4.025 Da (K). FDR of 1% was used to consider a positive identification.

### Quantification of ethanol content

Yeast cells in the exponential growth phase were incubated in SD medium with or without nitrite for 1 h. The ethanol concentration of supernatant was determined using Ethanol Colorimetric/Fluorometric Assay Kit (Biovision), following the manufacturer’s direction. Ethanol content in each sample was normalized by OD_600_ of the culture medium.

### Quantification of pyruvate content

Yeast cells cultured until the logarithmic phase were treated with the acidified nitrite for 1 h, harvested, and suspended in the pyruvate assay buffer, provided in Pyruvate Assay Kit (Sigma-Aldrich). Cell-free lysate extracted using Multi-beads shocker was centrifuged at 15,000 g for 15 min at 4 °C and then the supernatant was subjected to ultrafiltration with Amicon Ultra (10 KDa) (Merck), to remove insoluble materials and proteins. Pyruvate concentration in the resultant solution was measured using Pyruvate Assay Kit. Pyruvate content in each sample was normalized by OD_600_ of the culture medium.

## Supplementary Information

Supplementary Information.
