# Supplementary material for: Molecular mechanism of ethanol fermentation inhibition via protein tyrosine nitration of pyruvate decarboxylase by reactive nitrogen species in yeast
Source: Sci Rep. 2022 Mar 18;12:4664. doi: 10.1038/s41598-022-08568-4 (PMC8933545; doi:10.1038/s41598-022-08568-4)
Supplement: Supplementary file 1 — Supplementary Information. [file 41598_2022_8568_MOESM1_ESM.docx]

**Supplementary Information**

**Molecular Mechanism of Ethanol Fermentation Inhibition via Protein Tyrosine Nitration of Pyruvate Decarboxylase by Reactive Nitrogen Species in Yeast**

Supapid Eknikom^1,2^, Ryo Nasuno^1,2*^, Hiroshi Takagi^1ǂ^

^1^Division of Biological Science, Graduate School of Science and Technology, Nara Institute of Science and Technology, 8916-5, Takayama-cho, Ikoma, Nara 630-0192, Japan. ^2^These authors contributed equally: Supapid Eknikom and Ryo Nasuno. *Telephone: +81-743-72-5427; E-mail: r-nasuno@bs.naist.jp, ^ǂ^Telephone: +81-743-72-5420; E-mail: hiro@bs.naist.jp

Correspondence and requests for materials should be addressed to R.N. or H.T.

**Contents**

**Supplementary Figures S1-S7**

**Supplementary Tables S1-S10**

**Supplementary Figure 1**


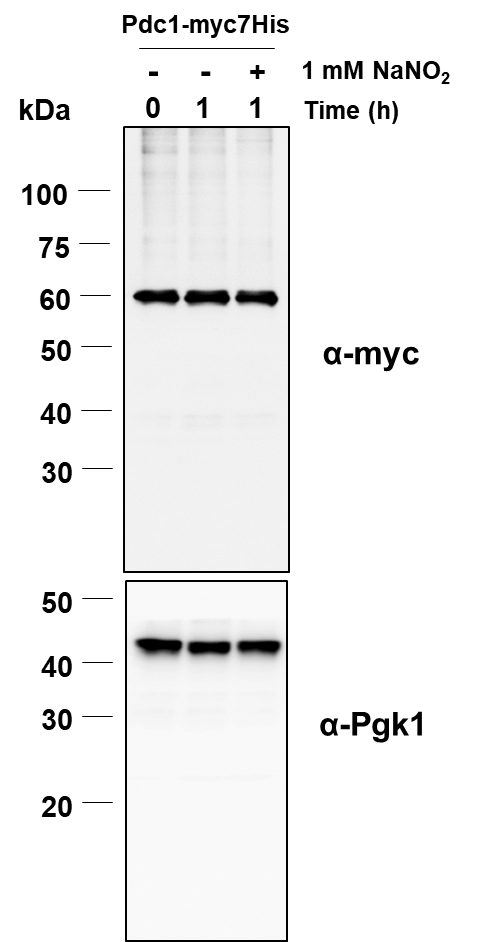


**Figure S1.** Protein levels of Pdc1 under the acidified nitrite treatment conditions. The PDC1-myc7His strain was treated with acidified nitrite and then the cell-free extracts were subjected to western blotting with anti-myc antibody, or anti-Pgk1 antibody as a loading control. The upper part of membrane for Pgk1 was cut out before the incubation with primary antibody.

**Supplementary Figure 2**


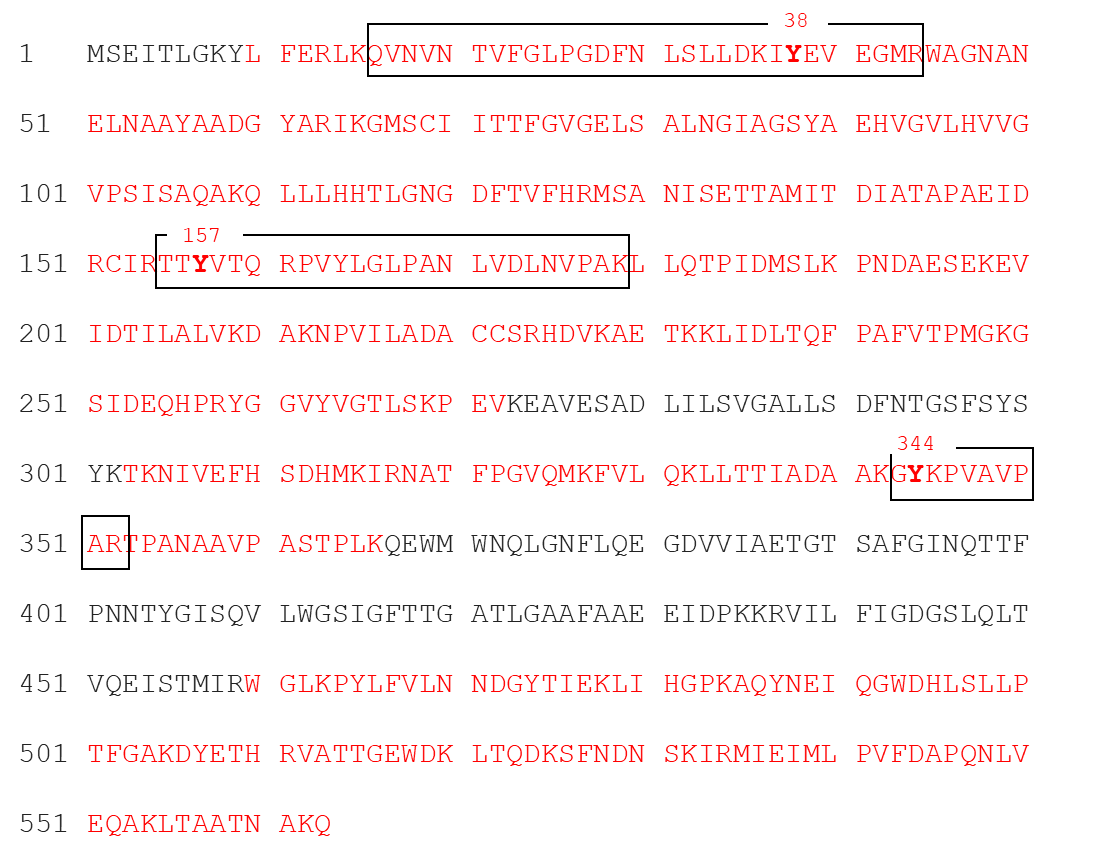


**Figure S2.** Peptide sequences of Pdc1 identified by LC-MS/MS. The amino acid sequence of Pdc1 from *S. cerevisiae* is shown. The sequences covered by identified peptides in the proteomic analysis were colored in red. Peptides identified as those containing AT were highlighted by black boxes. Each tyrosine residue detected with the AT modification was numbered.

**
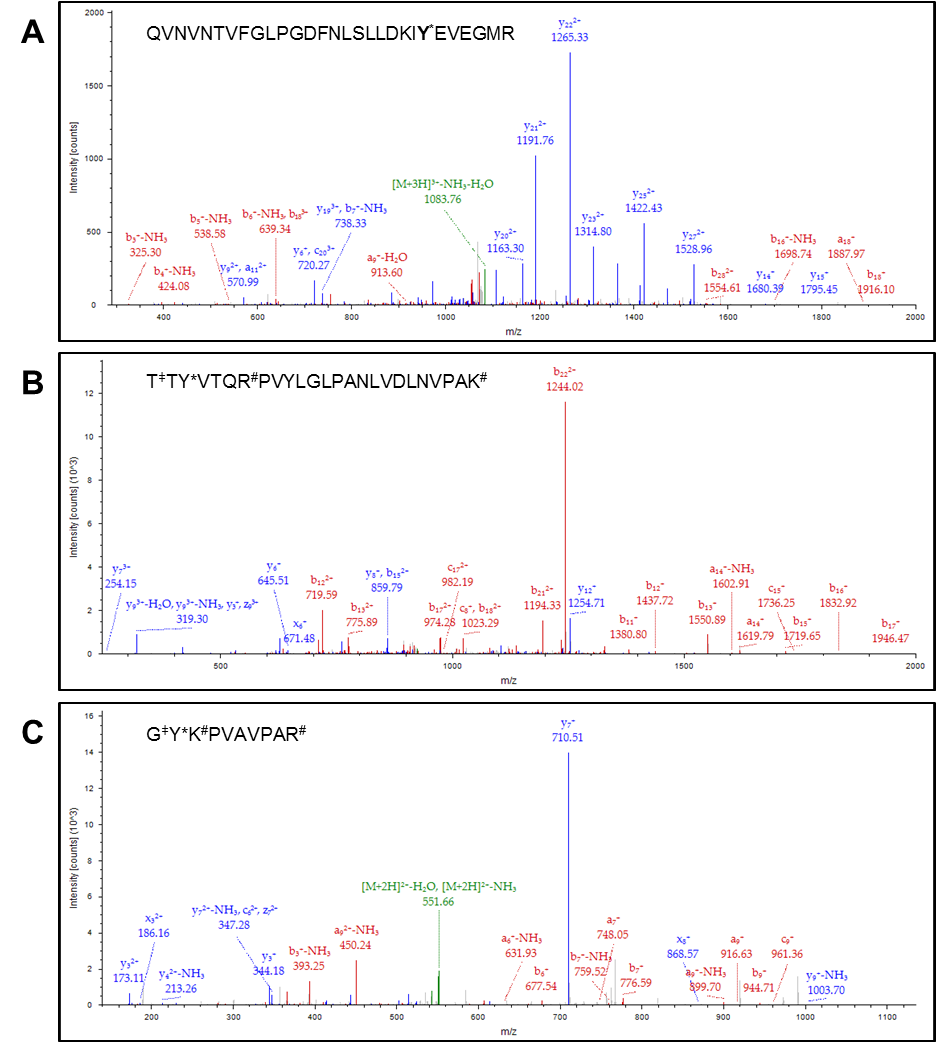
Supplementary Figure 3**

**Figure S3.** Annotated mass spectrum of Pdc1 peptide containing aminotyrosine. The MS/MS spectra for the identified peptides of Pdc1 containing AT as the trace of PTN at Tyr38 (A), Tyr157 (B), or Tyr344 (C), respectively, are shown. Peptide fragment ions containing the N-terminal portions (a, b, or c ion) or those containing the C-terminal portions (x, y, or z ion) are indicated in red or blue, respectively. The AT modification, N-terminal acetylation, or SILAC labeling of Lys and Arg are represented by *, #, or ǂ, respectively. All of the detected fragment ions for each peptide are listed in Table S1-S3.

**Supplementary Figure 4**

**
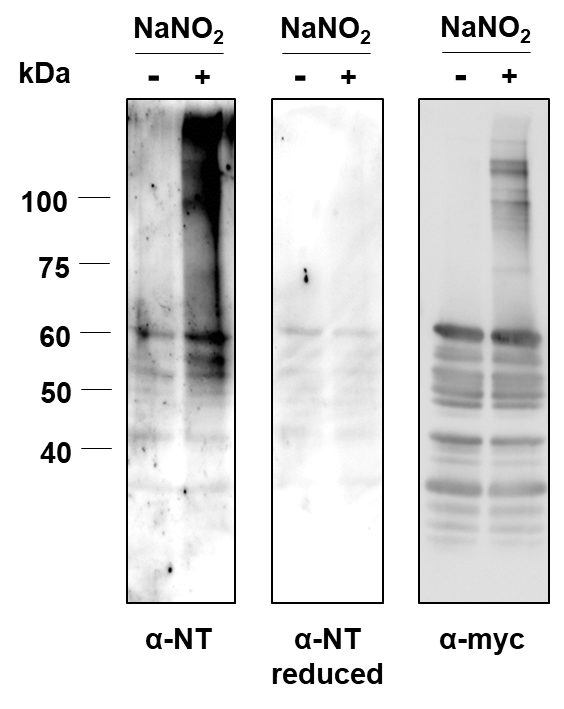
**

**Figure S4.** PTN modification of Pdc1 in response to RNS. Untrimmed membrane images corresponding to those in Figure 2 are shown.

**Supplementary Figure 5**

**
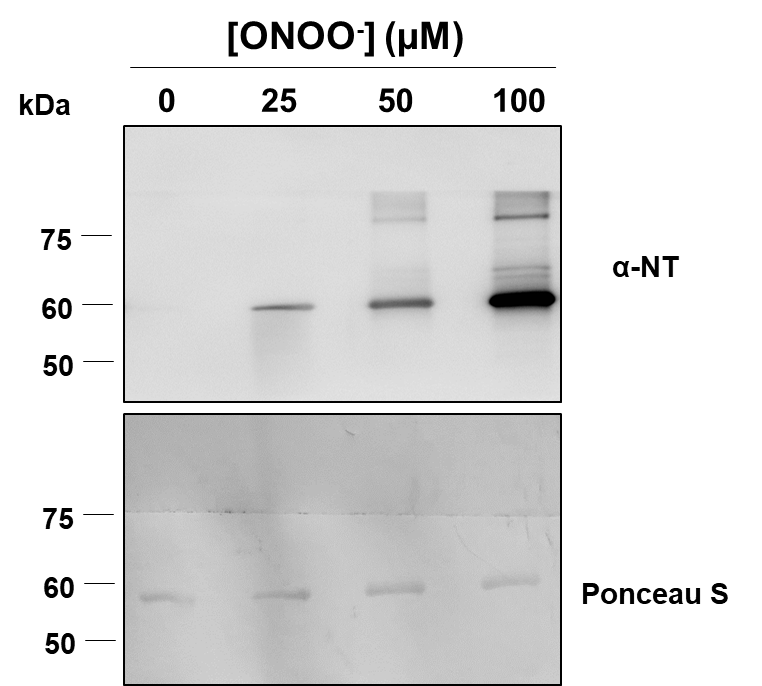
**

**Figure S5.** Immunoblotting of WT-Pdc1 treated with ONOO^-^ using anti-NT antibody. Untrimmed membrane images corresponding to those in Figure 3B are shown. The upper and lower parts of membranes were cut out before the incubation with primary antibody or Ponceau S.

**Supplementary Figure 6**


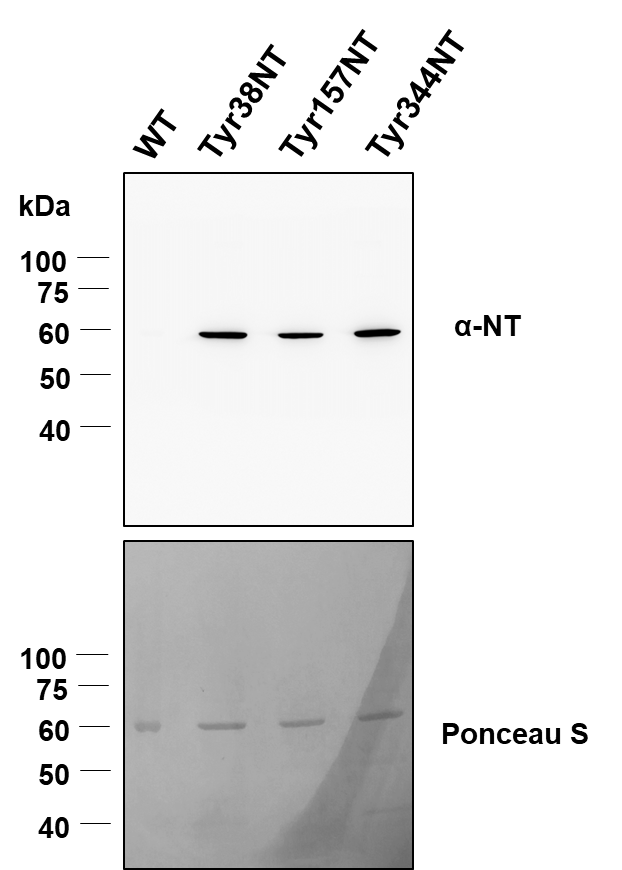


**Figure S6.** Immunoblotting of the recombinant Pdc1 site-specifically incorporating NT with anti-NT antibody. Untrimmed membrane images corresponding to those in Figure 3C are shown. The lower parts of membranes were cut out before the incubation with primary antibody or Ponceau S.

**Supplementary Figure 7**


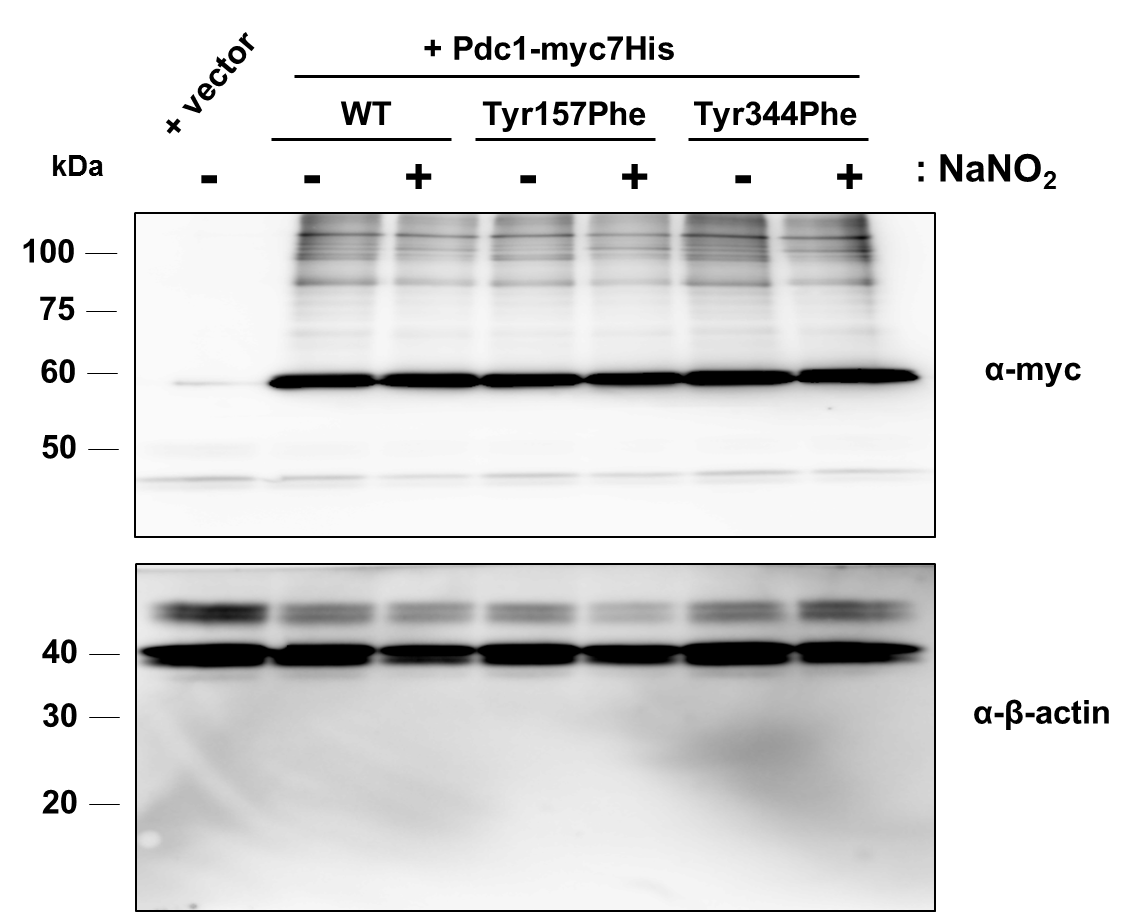


**Figure S7.** Immunoblotting of WT-, Tyr157Phe-, and Tyr344Phe-Pdc1 in response to RNS treatment. Untrimmed membrane images corresponding to those in Figure 4B are shown. The full membrane was cut away to the upper and lower parts for the detection of myc-tag-fused protein and β-actin, respectively, before the incubation with primary antibody.

**Table S1 Theoretically predicted and experimentally detected fragment ions from Tyr38-NH_2_-containing peptide.**

| **#1** | **immonium** | **a** | **b** | **c** | **Seq.** | **x** | **y** | **z** | **#2** |
| --- | --- | --- | --- | --- | --- | --- | --- | --- | --- |
| 1 | 101.07094 | 101.07094 | 129.06586 | 146.09241 | Q |  |  |  | 29 |
| 2 | 72.08078 | 200.13936 | 228.13428 | 245.16083 | V | 3180.60356 | 3154.62430 | 3138.60558 | 28 |
| 3 | 87.05529 | 314.18229 | 342.17721 | 359.20376 | N | 3081.53514 | 3055.55588 | 3039.53716 | 27 |
| 4 | 72.08078 | 413.25071 | 441.24563 | 458.27218 | V | 2967.49221 | 2941.51295 | 2925.49423 | 26 |
| 5 | 87.05529 | 527.29364 | 555.28856 | 572.31511 | N | 2868.42379 | 2842.44453 | 2826.42581 | 25 |
| 6 | 74.06004 | 628.34132 | 656.33624 | 673.36279 | T | 2754.38086 | 2728.40160 | 2712.38288 | 24 |
| 7 | 72.08078 | 727.40974 | 755.40466 | 772.43121 | V | 2653.33318 | 2627.35392 | 2611.33520 | 23 |
| 8 | 120.08078 | 874.47816 | 902.47308 | 919.49963 | F | 2554.26476 | 2528.28550 | 2512.26678 | 22 |
| 9 | 30.03383 | 931.49963 | 959.49455 | 976.52110 | G | 2407.19634 | 2381.21708 | 2365.19836 | 21 |
| 10 | 86.09643 | 1044.58370 | 1072.57862 | 1089.60517 | L | 2350.17487 | 2324.19561 | 2308.17689 | 20 |
| 11 | 70.06513 | 1141.63647 | 1169.63139 | 1186.65794 | P | 2237.09080 | 2211.11154 | 2195.09282 | 19 |
| 12 | 30.03383 | 1198.65794 | 1226.65286 | 1243.67941 | G | 2140.03803 | 2114.05877 | 2098.04005 | 18 |
| 13 | 88.03931 | 1313.68489 | 1341.67981 | 1358.70636 | D | 2083.01656 | 2057.03730 | 2041.01858 | 17 |
| 14 | 120.08078 | 1460.75331 | 1488.74823 | 1505.77478 | F | 1967.98961 | 1942.01035 | 1925.99163 | 16 |
| 15 | 87.05529 | 1574.79624 | 1602.79116 | 1619.81771 | N | 1820.92119 | 1794.94193 | 1778.92321 | 15 |
| 16 | 86.09643 | 1687.88031 | 1715.87523 | 1732.90178 | L | 1706.87826 | 1680.89900 | 1664.88028 | 14 |
| 17 | 60.04439 | 1774.91234 | 1802.90726 | 1819.93381 | S | 1593.79419 | 1567.81493 | 1551.79621 | 13 |
| 18 | 86.09643 | 1887.99641 | 1915.99133 | 1933.01788 | L | 1506.76216 | 1480.78290 | 1464.76418 | 12 |
| 19 | 86.09643 | 2001.08048 | 2029.07540 | 2046.10195 | L | 1393.67809 | 1367.69883 | 1351.68011 | 11 |
| 20 | 88.03931 | 2116.10743 | 2144.10235 | 2161.12890 | D | 1280.59402 | 1254.61476 | 1238.59604 | 10 |

**Table S1 Theoretically predicted and experimentally detected fragment ions from Tyr38-NH_2_-containing peptide (continued).**

| **#1** | **immonium** | **a** | **b** | **c** | **Seq.** | **x** | **y** | **z** | **#2** |
| --- | --- | --- | --- | --- | --- | --- | --- | --- | --- |
| 21 | 101.10733 | 2244.20240 | 2272.19732 | 2289.22387 | K | 1165.56707 | 1139.58781 | 1123.56909 | 9 |
| 22 | 86.09643 | 2357.28647 | 2385.28139 | 2402.30794 | I | 1037.47210 | 1011.49284 | 995.47412 | 8 |
| 23 | 136.07568 | 2535.36069 | 2563.35561 | 2580.38215 | Y-Amino | 924.38803 | 898.40877 | 882.39005 | 7 |
| 24 | 102.05496 | 2664.40329 | 2692.39821 | 2709.42475 | E | 746.31382 | 720.33455 | 704.31583 | 6 |
| 25 | 72.08078 | 2763.47171 | 2791.46663 | 2808.49317 | V | 617.27122 | 591.29195 | 575.27323 | 5 |
| 26 | 102.05496 | 2892.51431 | 2920.50923 | 2937.53577 | E | 518.20280 | 492.22353 | 476.20481 | 4 |
| 27 | 30.03383 | 2949.53578 | 2977.53070 | 2994.55724 | G | 389.16020 | 363.18093 | 347.16221 | 3 |
| 28 | 104.05286 | 3080.57628 | 3108.57120 | 3125.59774 | M | 332.13873 | 306.15946 | 290.14074 | 2 |
| 29 |  |  |  |  | R | 201.09823 | 175.11896 | 159.10024 | 1 |

Among the fragment ions theoretically predicted using Proteome Discoverer 1.4, the experimentally detected ones were highlighted in red.

**Table S2 Theoretically predicted and experimentally detected fragment ions from Tyr157-NH_2_-containing peptide.**

| **#1** | **immonium** | **a** | **b** | **c** | **Seq.** | **x** | **y** | **z** | **#2** |
| --- | --- | --- | --- | --- | --- | --- | --- | --- | --- |
| 1 | 74.06004 | 116.07061 | 144.06552 | 161.09207 | T-Acetyl |  |  |  | 25 |
| 2 | 74.06004 | 217.11829 | 245.11320 | 262.13975 | T | 2687.48993 | 2661.51066 | 2645.49194 | 24 |
| 3 | 136.07568 | 395.19251 | 423.18742 | 440.21397 | Y-Amino | 2586.44225 | 2560.46298 | 2544.44426 | 23 |
| 4 | 72.08078 | 494.26093 | 522.25584 | 539.28239 | V | 2408.36803 | 2382.38876 | 2366.37004 | 22 |
| 5 | 74.06004 | 595.30861 | 623.30352 | 640.33007 | T | 2309.29961 | 2283.32034 | 2267.30162 | 21 |
| 6 | 101.07094 | 723.36719 | 751.36210 | 768.38865 | Q | 2208.25193 | 2182.27266 | 2166.25394 | 20 |
| 7 | 129.11348 | 880.47166 | 908.46658 | 925.49312 | R-L-Arg(Guanido-^13^C) | 2080.19335 | 2054.21408 | 2038.19536 | 19 |
| 8 | 70.06513 | 977.52443 | 1005.51935 | 1022.54589 | P | 1923.08887 | 1897.10961 | 1881.09088 | 18 |
| 9 | 72.08078 | 1076.59285 | 1104.58777 | 1121.61431 | V | 1826.03610 | 1800.05684 | 1784.03811 | 17 |
| 10 | 136.07568 | 1239.65617 | 1267.65109 | 1284.67763 | Y | 1726.96768 | 1700.98842 | 1684.96969 | 16 |
| 11 | 86.09643 | 1352.74024 | 1380.73516 | 1397.76170 | L | 1563.90436 | 1537.92510 | 1521.90637 | 15 |
| 12 | 30.03383 | 1409.76171 | 1437.75663 | 1454.78317 | G | 1450.82029 | 1424.84103 | 1408.82230 | 14 |
| 13 | 86.09643 | 1522.84578 | 1550.84070 | 1567.86724 | L | 1393.79882 | 1367.81956 | 1351.80083 | 13 |
| 14 | 70.06513 | 1619.89855 | 1647.89347 | 1664.92001 | P | 1280.71475 | 1254.73549 | 1238.71676 | 12 |
| 15 | 44.04948 | 1690.93567 | 1718.93059 | 1735.95713 | A | 1183.66198 | 1157.68272 | 1141.66399 | 11 |
| 16 | 87.05529 | 1804.97860 | 1832.97352 | 1850.00006 | N | 1112.62486 | 1086.64560 | 1070.62687 | 10 |
| 17 | 86.09643 | 1918.06267 | 1946.05759 | 1963.08413 | L | 998.58193 | 972.60267 | 956.58394 | 9 |
| 18 | 72.08078 | 2017.13109 | 2045.12601 | 2062.15255 | V | 885.49786 | 859.51860 | 843.49987 | 8 |
| 19 | 88.03931 | 2132.15804 | 2160.15296 | 2177.17950 | D | 786.42944 | 760.45018 | 744.43145 | 7 |
| 20 | 86.09643 | 2245.24211 | 2273.23703 | 2290.26357 | L | 671.40249 | 645.42323 | 629.40450 | 6 |

**Table S2 Theoretically predicted and experimentally detected fragment ions from Tyr157-NH_2_-containing peptide (continued).**

| **#1** | **immonium** | **a** | **b** | **c** | **Seq.** | **x** | **y** | **z** | **#2** |
| --- | --- | --- | --- | --- | --- | --- | --- | --- | --- |
| 21 | 87.05529 | 2359.28504 | 2387.27996 | 2404.30650 | N | 558.31842 | 532.33916 | 516.32043 | 5 |
| 22 | 72.08078 | 2458.35346 | 2486.34838 | 2503.37492 | V | 444.27549 | 418.29623 | 402.27750 | 4 |
| 23 | 70.06513 | 2555.40623 | 2583.40115 | 2600.42769 | P | 345.20707 | 319.22781 | 303.20908 | 3 |
| 24 | 44.04948 | 2626.44335 | 2654.43827 | 2671.46481 | A | 248.15430 | 222.17504 | 206.15631 | 2 |
| 25 |  |  |  |  | K-Label:^2^H(4) | 177.11718 | 151.13792 | 135.11919 | 1 |

Among the fragment ions theoretically predicted using Proteome Discoverer 1.4, the experimentally detected ones were highlighted in red.

**Table S3 Theoretically predicted and experimentally detected fragment ions from Tyr344-NH_2_-containing peptide.**

| **#1** | **immonium** | **a** | **b** | **c** | **Seq.** | **x** | **y** | **z** | **#2** |
| --- | --- | --- | --- | --- | --- | --- | --- | --- | --- |
| 1 | 30.03383 | 72.04440 | 100.03931 | 117.06586 | G-Acetyl |  |  |  | 10 |
| 2 | 136.07568 | 250.11862 | 278.11353 | 295.14008 | Y-Amino | 1046.61250 | 1020.63323 | 1004.61451 | 9 |
| 3 | 101.10733 | 382.23869 | 410.23361 | 427.26016 | K-Label:^2^H(4) | 868.53828 | 842.55901 | 826.54029 | 8 |
| 4 | 70.06513 | 479.29146 | 507.28638 | 524.31293 | P | 736.41820 | 710.43894 | 694.42021 | 7 |
| 5 | 72.08078 | 578.35988 | 606.35480 | 623.38135 | V | 639.36543 | 613.38617 | 597.36744 | 6 |
| 6 | 44.04948 | 649.39700 | 677.39192 | 694.41847 | A | 540.29701 | 514.31775 | 498.29902 | 5 |
| 7 | 72.08078 | 748.46542 | 776.46034 | 793.48689 | V | 469.25989 | 443.28063 | 427.26190 | 4 |
| 8 | 70.06513 | 845.51819 | 873.51311 | 890.53966 | P | 370.19147 | 344.21221 | 328.19348 | 3 |
| 9 | 44.04948 | 916.55531 | 944.55023 | 961.57678 | A | 273.13870 | 247.15944 | 231.14071 | 2 |
| 10 |  |  |  |  | R-L-Arg(Guanido-^13^C) | 202.10158 | 176.12232 | 160.10359 | 1 |

Among the fragment ions theoretically predicted using Proteome Discoverer 1.4, the experimentally detected ones were highlighted in red.

**Table S4 Theoretically predicted fragment ions from Tyr38-NO_2_-containing peptide.**

| **#1** | **a** | **b** | **c** | **Seq.** | **x** | **y** | **z** | **#2** |
| --- | --- | --- | --- | --- | --- | --- | --- | --- |
| 1 | --- | --- | 146.0924 | Q | --- | --- | --- | 29 |
| 2 | 200.1394 | 228.1343 | 245.1608 | V | 3210.578 | 3184.598 | 3168.58 | 28 |
| 3 | 314.1823 | 342.1772 | 359.2037 | N | 3111.509 | 3085.53 | 3069.511 | 27 |
| 4 | 413.2507 | 441.2456 | 458.2722 | V | 2997.466 | 2971.487 | 2955.468 | 26 |
| 5 | 527.2936 | 555.2885 | 572.3151 | N | 2898.398 | 2872.419 | 2856.4 | 25 |
| 6 | 628.3413 | 656.3362 | 673.3628 | T | 2784.355 | 2758.376 | 2742.357 | 24 |
| 7 | 727.4097 | 755.4046 | 772.4312 | V | 2683.307 | 2657.328 | 2641.309 | 23 |
| 8 | 874.4781 | 902.473 | 919.4996 | F | 2584.239 | 2558.26 | 2542.241 | 22 |
| 9 | 931.4996 | 959.4945 | 976.5211 | G | 2437.17 | 2411.191 | 2395.172 | 21 |
| 10 | 1044.584 | 1072.579 | --- | L | 2380.149 | 2354.17 | 2338.151 | 20 |
| 11 | 1141.636 | 1169.631 | 1186.658 | P | 2267.065 | 2241.086 | --- | 19 |
| 12 | 1198.658 | 1226.653 | 1243.679 | G | 2170.012 | 2144.033 | 2128.014 | 18 |
| 13 | 1313.685 | 1341.68 | 1358.706 | D | 2112.991 | 2087.011 | 2070.993 | 17 |
| 14 | 1460.753 | 1488.748 | 1505.775 | F | 1997.964 | 1971.984 | 1955.966 | 16 |
| 15 | 1574.796 | 1602.791 | 1619.818 | N | 1850.895 | 1824.916 | 1808.897 | 15 |
| 16 | 1687.88 | 1715.875 | 1732.902 | L | 1736.852 | 1710.873 | 1694.854 | 14 |
| 17 | 1774.912 | 1802.907 | 1819.934 | S | 1623.768 | 1597.789 | 1581.77 | 13 |
| 18 | 1887.996 | 1915.991 | 1933.018 | L | 1536.736 | 1510.757 | 1494.738 | 12 |
| 19 | 2001.08 | 2029.075 | 2046.102 | L | 1423.652 | 1397.673 | 1381.654 | 11 |
| 20 | 2116.107 | 2144.102 | 2161.129 | D | 1310.568 | 1284.589 | 1268.57 | 10 |

**Table S4 Theoretically predicted fragment ions from Tyr38-NO_2_-containing peptide (continued).**

| **#1** | **a** | **b** | **c** | **Seq.** | **x** | **y** | **z** | **#2** |
| --- | --- | --- | --- | --- | --- | --- | --- | --- |
| 21 | 2244.202 | 2272.197 | 2289.224 | K | 1195.541 | 1169.562 | 1153.543 | 9 |
| 22 | 2357.286 | 2385.281 | 2402.308 | I | 1067.446 | 1041.467 | 1025.448 | 8 |
| 23 | 2565.335 | 2593.33 | 2610.356 | Y-Nitro | 954.3622 | 928.3829 | 912.3642 | 7 |
| 24 | 2694.377 | 2722.372 | 2739.399 | E | 746.3138 | 720.3345 | 704.3158 | 6 |
| 25 | 2793.446 | 2821.441 | 2838.467 | V | 617.2712 | 591.2919 | 575.2732 | 5 |
| 26 | 2922.488 | 2950.483 | 2967.51 | E | 518.2028 | 492.2235 | 476.2048 | 4 |
| 27 | 2979.51 | 3007.505 | 3024.531 | G | 389.1602 | 363.1809 | 347.1622 | 3 |
| 28 | 3110.55 | 3138.545 | 3155.572 | M | 332.1387 | 306.1594 | 290.1407 | 2 |
| 29 | --- | --- | --- | R | 201.0982 | 175.119 | 159.1002 | 1 |

The theoretical fragmentations of the peptide containing Tyr38-NO_2_, which corresponds to that shown in Table S1, were predicted using a proteomic web tool MS-product in ProteinProspector (https://prospector.ucsf.edu/prospector/cgi-bin/msform.cgi?form=msproduct).

**Table S5 Theoretically predicted fragment ions from Tyr157-NO_2_-containing peptide.**

| **#1** | **a** | **b** | **c** | **Seq.** | **x** | **y** | **z** | **#2** |
| --- | --- | --- | --- | --- | --- | --- | --- | --- |
| 1 | 116.0706 | 144.0655 | 161.0921 | T-Acetyl | --- | 2792.532 | --- | 25 |
| 2 | 217.1183 | 245.1132 | 262.1397 | T | 2717.464 | 2691.485 | 2675.466 | 24 |
| 3 | 425.1667 | 453.1616 | 470.1882 | Y-Nitro | 2616.416 | 2590.437 | 2574.418 | 23 |
| 4 | 524.2351 | 552.23 | 569.2566 | V | 2408.368 | 2382.389 | 2366.37 | 22 |
| 5 | 625.2828 | 653.2777 | 670.3042 | T | 2309.3 | 2283.32 | 2267.302 | 21 |
| 6 | 753.3414 | 781.3363 | 798.3628 | Q | 2208.252 | 2182.273 | 2166.254 | 20 |
| 7 | 910.4458 | 938.4407 | --- | R-L-Arg(Guanido-^13^C) | 2080.193 | 2054.214 | 2038.195 | 19 |
| 8 | 1007.499 | 1035.494 | 1052.52 | P | 1923.089 | 1897.11 | --- | 18 |
| 9 | 1106.567 | 1134.562 | 1151.589 | V | 1826.036 | 1800.057 | 1784.038 | 17 |
| 10 | 1269.63 | 1297.625 | 1314.652 | Y | 1726.968 | 1700.988 | 1684.97 | 16 |
| 11 | 1382.714 | 1410.709 | 1427.736 | L | 1563.904 | 1537.925 | 1521.906 | 15 |
| 12 | 1439.736 | 1467.731 | 1484.757 | G | 1450.82 | 1424.841 | 1408.822 | 14 |
| 13 | 1552.82 | 1580.815 | --- | L | 1393.799 | 1367.82 | 1351.801 | 13 |
| 14 | 1649.873 | 1677.868 | 1694.894 | P | 1280.715 | 1254.735 | --- | 12 |
| 15 | 1720.91 | 1748.905 | 1765.931 | A | 1183.662 | 1157.683 | 1141.664 | 11 |
| 16 | 1834.953 | 1862.948 | 1879.974 | N | 1112.625 | 1086.646 | 1070.627 | 10 |
| 17 | 1948.037 | 1976.032 | 1993.058 | L | 998.5819 | 972.6026 | 956.5839 | 9 |
| 18 | 2047.105 | 2075.1 | 2092.127 | V | 885.4978 | 859.5186 | 843.4998 | 8 |
| 19 | 2162.132 | 2190.127 | 2207.154 | D | 786.4294 | 760.4501 | 744.4314 | 7 |
| 20 | 2275.216 | 2303.211 | 2320.238 | L | 671.4025 | 645.4232 | 629.4045 | 6 |

**Table S5 Theoretically predicted fragment ions from Tyr157-NO_2_-containing peptide (continued).**

| **#1** | **a** | **b** | **c** | **Seq.** | **x** | **y** | **z** | **#2** |
| --- | --- | --- | --- | --- | --- | --- | --- | --- |
| 21 | 2389.259 | 2417.254 | 2434.281 | N | 558.3184 | 532.3391 | 516.3204 | 5 |
| 22 | 2488.328 | 2516.323 | --- | V | 444.2755 | 418.2962 | 402.2775 | 4 |
| 23 | 2585.38 | 2613.375 | 2630.402 | P | 345.2071 | 319.2278 | --- | 3 |
| 24 | 2656.417 | 2684.412 | 2701.439 | A | 248.1543 | 222.175 | 206.1563 | 2 |
| 25 | --- | --- | --- | K-Label:^2^H(4) | 177.1172 | 151.1379 | 135.1192 | 1 |

The theoretical fragmentations of the peptide containing Tyr157-NO_2_, which corresponds to that shown in Table S2, were predicted using a proteomic web tool MS-product in ProteinProspector (https://prospector.ucsf.edu/prospector/cgi-bin/msform.cgi?form=msproduct).

**Table S6 Theoretically predicted fragment ions from Tyr344-NO_2_-containing peptide.**

| **#1** | **a** | **b** | **c** | **Seq.** | **x** | **y** | **z** | **#2** |
| --- | --- | --- | --- | --- | --- | --- | --- | --- |
| **1** | 72.0444 | 100.0393 | 117.0659 | G-Acetyl | --- | 1107.629 | --- | **10** |
| **2** | 280.0928 | 308.0877 | 325.1143 | Y-Nitro | 1076.587 | 1050.607 | 1034.589 | **9** |
| **3** | 412.2129 | 440.2078 | --- | K-Label:^2^H(4) | 868.5382 | 842.559 | 826.5402 | **8** |
| **4** | 509.2656 | 537.2605 | 554.2871 | P | 736.4182 | 710.4389 | --- | **7** |
| **5** | 608.334 | 636.329 | 653.3555 | V | 639.3654 | 613.3861 | 597.3674 | **6** |
| **6** | 679.3712 | 707.3661 | 724.3926 | A | 540.297 | 514.3177 | 498.299 | **5** |
| **7** | 778.4396 | 806.4345 | --- | V | 469.2599 | 443.2806 | 427.2619 | **4** |
| **8** | 875.4923 | 903.4873 | 920.5138 | P | 370.1914 | 344.2122 | --- | **3** |
| **9** | 946.5295 | 974.5244 | 991.5509 | A | 273.1387 | 247.1594 | 231.1407 | **2** |
| **10** | --- | --- | --- | R-L-Arg(Guanido-^13^C) | 202.1016 | 176.1223 | 160.1036 | **1** |

The theoretical fragmentations of the peptide containing Tyr344-NO_2_, which corresponds to that shown in Table S3, were predicted using a proteomic web tool MS-product in ProteinProspector (https://prospector.ucsf.edu/prospector/cgi-bin/msform.cgi?form=msproduct).

**Table S7 Theoretically predicted and experimentally detected fragment ions from non-modified Tyr38-containing peptide.**

| **#1** | **immonium** | **a** | **b** | **c** | **Seq.** | **x** | **y** | **z** | **#2** |
| --- | --- | --- | --- | --- | --- | --- | --- | --- | --- |
| **1** | 101.07094 | 101.07094 | 129.06586 | 146.09241 | Q |  |  |  | **29** |
| **2** | 72.08078 | 200.13936 | 228.13428 | 245.16083 | V | 3181.58758 | 3155.60832 | 3139.58959 | **28** |
| **3** | 87.05529 | 314.18229 | 342.17721 | 359.20376 | N | 3082.51916 | 3056.53990 | 3040.52117 | **27** |
| **4** | 72.08078 | 413.25071 | 441.24563 | 458.27218 | V | 2968.47623 | 2942.49697 | 2926.47824 | **26** |
| **5** | 87.05529 | 527.29364 | 555.28856 | 572.31511 | N | 2869.40781 | 2843.42855 | 2827.40982 | **25** |
| **6** | 74.06004 | 628.34132 | 656.33624 | 673.36279 | T | 2755.36488 | 2729.38562 | 2713.36689 | **24** |
| **7** | 72.08078 | 727.40974 | 755.40466 | 772.43121 | V | 2654.31720 | 2628.33794 | 2612.31921 | **23** |
| **8** | 120.08078 | 874.47816 | 902.47308 | 919.49963 | F | 2555.24878 | 2529.26952 | 2513.25079 | **22** |
| **9** | 30.03383 | 931.49963 | 959.49455 | 976.52110 | G | 2408.18036 | 2382.20110 | 2366.18237 | **21** |
| **10** | 86.09643 | 1044.58370 | 1072.57862 | 1089.60517 | L | 2351.15889 | 2325.17963 | 2309.16090 | **20** |
| **11** | 70.06513 | 1141.63647 | 1169.63139 | 1186.65794 | P | 2238.07482 | 2212.09556 | 2196.07683 | **19** |
| **12** | 30.03383 | 1198.65794 | 1226.65286 | 1243.67941 | G | 2141.02205 | 2115.04279 | 2099.02406 | **18** |
| **13** | 88.03931 | 1313.68489 | 1341.67981 | 1358.70636 | D | 2084.00058 | 2058.02132 | 2042.00259 | **17** |
| **14** | 120.08078 | 1460.75331 | 1488.74823 | 1505.77478 | F | 1968.97363 | 1942.99437 | 1926.97564 | **16** |
| **15** | 87.05529 | 1574.79624 | 1602.79116 | 1619.81771 | N | 1821.90521 | 1795.92595 | 1779.90722 | **15** |
| **16** | 86.09643 | 1687.88031 | 1715.87523 | 1732.90178 | L | 1707.86228 | 1681.88302 | 1665.86429 | **14** |
| **17** | 60.04439 | 1774.91234 | 1802.90726 | 1819.93381 | S | 1594.77821 | 1568.79895 | 1552.78022 | **13** |
| **18** | 86.09643 | 1887.99641 | 1915.99133 | 1933.01788 | L | 1507.74618 | 1481.76692 | 1465.74819 | **12** |
| **19** | 86.09643 | 2001.08048 | 2029.07540 | 2046.10195 | L | 1394.66211 | 1368.68285 | 1352.66412 | **11** |
| **20** | 88.03931 | 2116.10743 | 2144.10235 | 2161.12890 | D | 1281.57804 | 1255.59878 | 1239.58005 | **10** |

**Table S7 Theoretically predicted and experimentally detected fragment ions from non-modified Tyr38-containing peptide (continued).**

| **#1** | **immonium** | **a** | **b** | **c** | **Seq.** | **x** | **y** | **z** | **#2** |
| --- | --- | --- | --- | --- | --- | --- | --- | --- | --- |
| **21** | 101.10733 | 2244.20240 | 2272.19732 | 2289.22387 | K | 1166.55109 | 1140.57183 | 1124.55310 | **9** |
| **22** | 86.09643 | 2357.28647 | 2385.28139 | 2402.30794 | I | 1038.45612 | 1012.47686 | 996.45813 | **8** |
| **23** | 136.07568 | 2520.34979 | 2548.34471 | 2565.37126 | Y | 925.37205 | 899.39279 | 883.37406 | **7** |
| **24** | 102.05496 | 2649.39239 | 2677.38731 | 2694.41386 | E | 762.30873 | 736.32947 | 720.31074 | **6** |
| **25** | 72.08078 | 2748.46081 | 2776.45573 | 2793.48228 | V | 633.26613 | 607.28687 | 591.26814 | **5** |
| **26** | 102.05496 | 2877.50341 | 2905.49833 | 2922.52488 | E | 534.19771 | 508.21845 | 492.19972 | **4** |
| **27** | 30.03383 | 2934.52488 | 2962.51980 | 2979.54635 | G | 405.15511 | 379.17585 | 363.15712 | **3** |
| **28** | 104.05286 | 3081.56030 | 3109.55521 | 3126.58176 | M-Oxidation | 348.13364 | 322.15438 | 306.13565 | **2** |
| **29** |  |  |  |  | R | 201.09823 | 175.11896 | 159.10024 | **1** |

Among the fragment ions theoretically predicted using Proteome Discoverer 1.4, the experimentally detected ones were highlighted in red.

**Table S8 Theoretically predicted and experimentally detected fragment ions from non-modified Tyr157-containing peptide.**

| **#1** | **immonium** | **a** | **b** | **c** | **Seq.** | **x** | **y** | **z** | **#2** |
| --- | --- | --- | --- | --- | --- | --- | --- | --- | --- |
| **1** | 74.06004 | 74.06004 | 102.05496 | 119.08151 | T |  |  |  | **25** |
| **2** | 74.06004 | 175.10772 | 203.10264 | 220.12919 | T | 2672.47903 | 2646.49976 | 2630.48104 | **24** |
| **3** | 136.07568 | 338.17104 | 366.16596 | 383.19251 | Y | 2571.43135 | 2545.45208 | 2529.43336 | **23** |
| **4** | 72.08078 | 437.23946 | 465.23438 | 482.26093 | V | 2408.36803 | 2382.38876 | 2366.37004 | **22** |
| **5** | 74.06004 | 538.28714 | 566.28206 | 583.30861 | T | 2309.29961 | 2283.32034 | 2267.30162 | **21** |
| **6** | 101.07094 | 666.34572 | 694.34064 | 711.36719 | Q | 2208.25193 | 2182.27266 | 2166.25394 | **20** |
| **7** | 129.11348 | 823.45020 | 851.44511 | 868.47166 | R-L-Arg(Guanido-^13^C) | 2080.19335 | 2054.21408 | 2038.19536 | **19** |
| **8** | 70.06513 | 920.50297 | 948.49788 | 965.52443 | P | 1923.08887 | 1897.10961 | 1881.09088 | **18** |
| **9** | 72.08078 | 1019.57139 | 1047.56630 | 1064.59285 | V | 1826.03610 | 1800.05684 | 1784.03811 | **17** |
| **10** | 136.07568 | 1182.63471 | 1210.62962 | 1227.65617 | Y | 1726.96768 | 1700.98842 | 1684.96969 | **16** |
| **11** | 86.09643 | 1295.71878 | 1323.71369 | 1340.74024 | L | 1563.90436 | 1537.92510 | 1521.90637 | **15** |
| **12** | 30.03383 | 1352.74025 | 1380.73516 | 1397.76171 | G | 1450.82029 | 1424.84103 | 1408.82230 | **14** |
| **13** | 86.09643 | 1465.82432 | 1493.81923 | 1510.84578 | L | 1393.79882 | 1367.81956 | 1351.80083 | **13** |
| **14** | 70.06513 | 1562.87709 | 1590.87200 | 1607.89855 | P | 1280.71475 | 1254.73549 | 1238.71676 | **12** |
| **15** | 44.04948 | 1633.91421 | 1661.90912 | 1678.93567 | A | 1183.66198 | 1157.68272 | 1141.66399 | **11** |
| **16** | 87.05529 | 1747.95714 | 1775.95205 | 1792.97860 | N | 1112.62486 | 1086.64560 | 1070.62687 | **10** |
| **17** | 86.09643 | 1861.04121 | 1889.03612 | 1906.06267 | L | 998.58193 | 972.60267 | 956.58394 | **9** |
| **18** | 72.08078 | 1960.10963 | 1988.10454 | 2005.13109 | V | 885.49786 | 859.51860 | 843.49987 | **8** |
| **19** | 88.03931 | 2075.13658 | 2103.13149 | 2120.15804 | D | 786.42944 | 760.45018 | 744.43145 | **7** |
| **20** | 86.09643 | 2188.22065 | 2216.21556 | 2233.24211 | L | 671.40249 | 645.42323 | 629.40450 | **6** |

**Table S8 Theoretically predicted and experimentally detected fragment ions from non-modified Tyr157-containing peptide (continued).**

| **#1** | **immonium** | **a** | **b** | **c** | **Seq.** | **x** | **y** | **z** | **#2** |
| --- | --- | --- | --- | --- | --- | --- | --- | --- | --- |
| **21** | 87.05529 | 2302.26358 | 2330.25849 | 2347.28504 | N | 558.31842 | 532.33916 | 516.32043 | **5** |
| **22** | 72.08078 | 2401.33200 | 2429.32691 | 2446.35346 | V | 444.27549 | 418.29623 | 402.27750 | **4** |
| **23** | 70.06513 | 2498.38477 | 2526.37968 | 2543.40623 | P | 345.20707 | 319.22781 | 303.20908 | **3** |
| **24** | 44.04948 | 2569.42189 | 2597.41680 | 2614.44335 | A | 248.15430 | 222.17504 | 206.15631 | **2** |
| **25** |  |  |  |  | K-Label:^2^H(4) | 177.11718 | 151.13792 | 135.11919 | **1** |

Among the fragment ions theoretically predicted using Proteome Discoverer 1.4, the experimentally detected ones were highlighted in red.

**Table S9 Theoretically predicted and experimentally detected fragment ions from non-modified Tyr344-containing peptide.**

| **#1** | **immonium** | **a** | **b** | **c** | **Seq.** | **x** | **y** | **z** | **#2** |
| --- | --- | --- | --- | --- | --- | --- | --- | --- | --- |
| **1** | 30.03383 | 30.03383 | 58.02875 | 75.05530 | G |  |  |  | **10** |
| **2** | 136.07568 | 193.09715 | 221.09207 | 238.11862 | Y | 1031.60160 | 1005.62233 | 989.60361 | **9** |
| **3** | 101.10733 | 325.21723 | 353.21214 | 370.23869 | K-Label:^2^H(4) | 868.53828 | 842.55901 | 826.54029 | **8** |
| **4** | 70.06513 | 422.27000 | 450.26491 | 467.29146 | P | 736.41820 | 710.43894 | 694.42021 | **7** |
| **5** | 72.08078 | 521.33842 | 549.33333 | 566.35988 | V | 639.36543 | 613.38617 | 597.36744 | **6** |
| **6** | 44.04948 | 592.37554 | 620.37045 | 637.39700 | A | 540.29701 | 514.31775 | 498.29902 | **5** |
| **7** | 72.08078 | 691.44396 | 719.43887 | 736.46542 | V | 469.25989 | 443.28063 | 427.26190 | **4** |
| **8** | 70.06513 | 788.49673 | 816.49164 | 833.51819 | P | 370.19147 | 344.21221 | 328.19348 | **3** |
| **9** | 44.04948 | 859.53385 | 887.52876 | 904.55531 | A | 273.13870 | 247.15944 | 231.14071 | **2** |
| **10** |  |  |  |  | R-L-Arg(Guanido-^13^C) | 202.10158 | 176.12232 | 160.10359 | **1** |

Among the fragment ions theoretically predicted using Proteome Discoverer 1.4, the experimentally detected ones were highlighted in red.

**Table S10. Oligonucleotide primers used in this study.**

| Primer | Sequences (5’-3’) | Description |
| --- | --- | --- |
| *PDC1_*S1_Fw | TTATTTTCTACTCATAACCTCACGCAAAATAACACAGTCAAATCAATCAA AATGCGTACGCTGCAGGTCGAC | Deletion of *PDC1* |
| *PDC1_*S2_Rv | GTTACATAAAAATGCTTATAAAACTTTAACTAATAATTAGAGATTAAATCGCTTAATCGATGAATTCGAGCTCG | Deletion of *PDC1*/ Fusion of Pdc1 with a tag |
| *PDC1_*S3_Fw | TCCACAAAACTTGGTTGAACAAGCTAAGTTGACTGCTGCTACCAACGCTAAGCAACGTACGCTGCAGGTCGAC | Fusion of Pdc1 with a tag |
| PDC1_ Up1000_Fw | CGGGCCCCCCCTCGAAAAATGAAGGCCAAATCAAGGCGG | Construction of the plasmid expressing Pdc1-myc7His in yeast |
| PDC1_Rv | CGGGCTGCAGGAATTGGTAGAGGTGTGGTCAATAAGAGCG |  |
| attB1-*PDC1_*Fw | GGGGACAAGTTTGTACAAAAAAGCAGGCTTCTCTGAAATTACTTTGGGTAAATATTTG | Construction of pET55-PDC1 |
| attB2-*PDC1_*Rv | GGGGACCACTTTGTACAAGAAAGCTGGGTGTTGCTTAGCGTTGGTAGCAGCAGT |  |
| Arg1_dis_S1_Fw | TAATATACACGGATACAAAAGAAATACACATAATTGCATAAAATACGTACGCTGCAGGTCGAC | Deletion of *ARG1* |
| Arg1_dis_S2_Rv | GGAGGAAGAGATCGTTATCTATCTTGAGGCGATGAACTAGCGGACATCGATGAATTCGAGCTCG |  |

**Table S10. Oligonucleotide primers used in this study (continued).**

| Primer | Sequences (5’-3’) | Description |
| --- | --- | --- |
| Lys1_dis_S1_Fw | ATAAGATAACAACGAAAACGCTTTATTTTTCACACAACCGCAAAACGTACGCTGCAGGTCGAC | Deletion of *LYS1* |
| Lys1_dis_S2_Rv | TTGTAAATGTCAGCGTAACGATAATGTATATACTTTAAATGTAAAATCGATGAATTCGAGCTCG |  |
| Y38NT_Fw | ATCTCATACCTTCAACTTCCTAGATCTTGTCCAACAAGG | Amino acid substitution (Tyr38NT) |
| Y38NT_Rv | CCTTGTTGGACAAGATCTAGGAAGTTGAAGGTATGAGAT |  |
| Y157NT_Fw | CTGGTCTTTGGGTGACCTAAGTGGTTCTGATACA | Amino acid substitution (Tyr157NT) |
| Y157NT_Rv | TGTATCAGAACCACTTAGGTCACCCAAAGACCAG |  |
| Y344NT_Fw | AGCAACTGGCTTCTAACCCTTAGCGGCGTC | Amino acid substitution (Tyr344NT) |
| Y344NT_Rv | GACGCCGCTAAGGGTTAGAAGCCAGTTGCT |  |
| Y157F_Fw | GGTCTTTGGGTGACGAAAGTGGTTCTGATACAT | Amino acid substitution (Tyr157Phe) |
| Y157F_Rv | ATGTATCAGAACCACTTTCGTCACCCAAAGACC |  |
| Y344F_Fw | AGCAACTGGCTTGAAACCCTTAGCGGC | Amino acid substitution (Tyr344Phe) |
| Y344F_Rv | GCCGCTAAGGGTTTCAAGCCAGTTGCT |  |
